# Notes and News

**Published:** 1893-02-04

**Authors:** 


### Notes and News.

Collected and Communicated.

We are requested to announce that a meeting of practitioners will be held at the rooms of Dr. George W. Potter, 8, King Street, Cheapside, on Monday, February 6th, at four p.m., for the purpose of constituting a Central London Branch of the London and Counties Medical Protection Society. Dr. G. B. Mead, the Organising Secretary, will be present, and registered practitioners in the district are invited to attend.

The recommendation of the Lords' Committee to establish further general hospital accommodation in South-East London is progressing. A special general meeting of the Miller Memorial Hospital is to be held in the board-room of the Dispensary, Greenwich Road, on Monday, February 6th, at eight p.m., when the further extension of the hospital will be considered in accordance with a scheme prepared by the sub-committee appointed by the governors.

The National Society for the Employment of Epileptics held a large meeting at the Mansion House last week, under the presidency of the Lord Mayor, who was supported by Sir Andrew Clark, Sir J. Crichton-Brown, and many other influential persons. The object of the Society is to establish a Colony for Epileptics, with which scheme all must heartily sympathise. Such undertakings have proved most successful on the Continent in effecting the object for which they were started, but it can never be hoped that they will be entirely self-supporting. The National Society only propose to establish their colony on one hundred acres of land near London. This land Mr. Passmore Edwards has most generously promised to provide. Whilst heartily approving of the proposal of an epileptic colony, we feel that the scheme is being started on altogether too small a scale for anything which should be regarded as a national movement. That so small a sum as £10,000 is asked for is altogether misleading. Once alive to the necessities of the case, the nation would be found willing to provide ten times such a sum. We do not hesitate to say this, as the example of one English woman, Lady Meath, who has expended £8,000 on her Home for Epileptics at Godalming, goes to show how deep is the sympathy of those who really know the needs of this most afflicted class.

We are glad to be able to draw our readers' attention to some excellent articles in "The Trained Nurse" for January, on the subject of the care of the eyes, especially of those of very young children. Special stress is laid, in these articles, on the preventable nature of ophthalmia amongst infants, and many valuable hints are given.

Fires in London have, we learn from the report of the Fire Brigade, been more numerous by 254 in the past year than they were in 1891. The work of this most useful institution can be better understood when we learn that one year of fires entails 34,350 journeys for the Brigade. It is greatly to the discredit of the populace that as many as 491 false alarms were received during the year, as the hard-worked Brigade are a body who confer infinite service to the whole community and are most deservedly respected and popular.

It is not at all unusual to find the provinces in the van of progressive measures of all kinds. The Sheffield Guardians made an important step forward on the road of civilisation when last week they adopted the scheme for dividing the deserving from the undeserving paupers in a classification embracing four classes. The first class is to contain those who, over sixty years of age, have lived twenty years in the township without reproach, and without having received relief. The second class comprises those who have fallen slightly short of these qualifications. The third class will contain persons of an inferior, and the fourth those of decidedly bad character. The better classes are to be provided with superior diet, accommodation, clothing, and other advantages.

We welcome the *Westminster Gazette* as an old but sturdy opponent with a new face. It is distinctly to the advantage of London and Londoners that Liberal principles should be adequately represented in the metropolitan evening press. Londoners know by past experience that the present conductors of the *Westminster Gazette* will worthily represent the best type of Liberalism, whilst their courageous independence on a recent occasion has won for them the respect and sympathy of every responsible journalist. The *Westminster Gazette* has assumed a colour which will make the paper specially interesting to the profession. We have carefully tested this paper, the colour of which has been selected "to minimise the the difficulties arising from badly lighted railway carriages and omnibuses." Judging by the tests applied, we have formed the conclusion that the *Westminster Gazette* will be the most widely read of evening newspapers, for whereas in daylight the paper and type are distinctly less legible than those printed in the orthodox way, by artificial light the new colour proves most helpful and restful to the reader's eye. How would it be for the *Westminster Gazette* to determine to reverse the order of nature by assuming a green garb during the autumn and winter and to exchange it for white during the spring and summer? Anyhow, our new contemporary is heartily welcome, and deserves the sympathetic support of all Londoners, quite apart from any question of politics.

